# Transcatheter aortic valve replacement valve endocarditis requiring Commando procedure

**DOI:** 10.1016/j.xjtc.2024.06.011

**Published:** 2024-06-28

**Authors:** Omar A. Jarral, Stevan S. Pupovac, Jui-Chuan Tseng, Chad A. Kliger, Luigi Pirelli, Kush R. Dholakia, Nirav C. Patel, S. Jacob Scheinerman, Alan R. Hartman, Derek R. Brinster

**Affiliations:** Department of Cardiovascular and Thoracic Surgery, Northwell Cardiovascular Institute, New Hyde Park, NY

**Figure:**
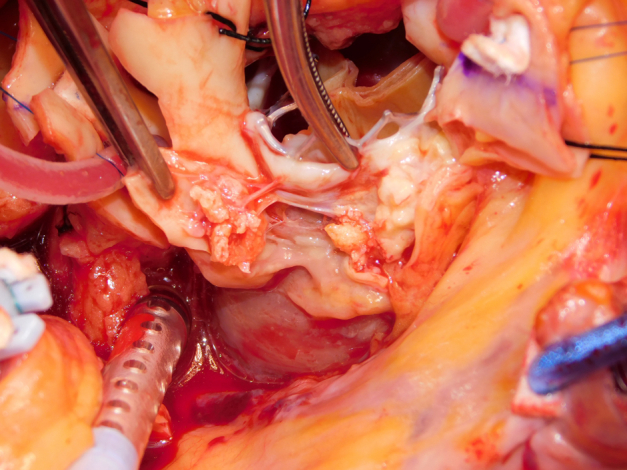
Transcatheter valve endocarditis with extensive destruction of the aortomitral curtain.

**Central Message**

TAVR explant is the fastest growing cardiac surgical operation. Some cases will require radical debridement of the aortomitral curtain. A sound understanding of the Commando procedure is required.

Because transcatheter aortic valve replacement explant is the fastest growing operation in the Society of Thoracic Surgeons database,^1^ it is essential that surgeons carrying out this procedure have an understanding of the principles underlying the Commando procedure, should it be required.

A 64-year-old man who previously underwent transcatheter aortic valve replacement presented with a 2-week history of fever. Investigations demonstrated aortic (AV) and mitral valve (MV) endocarditis, with extensive root abscess. After sternotomy/routine setup, the AV was exposed with aortotomy extending into the noncoronary sinus. Radical debridement was performed, with incision into the left atrial roof (Video 1 and Figure 1). Interrupted sutures were placed in the posterior mitral annulus. A 29-mm biological prosthesis was implanted (Figure 2). The AV was sized. A double patch of bovine pericardium was sutured starting from the lateral trigone, in an anticlockwise direction to reconstruct the dome of the atrium. The other end of the suture was used to run along the mitral prosthesis to the medial trigone. Interrupted sutures for the root were placed, and the conduit was secured. A modified Cabrol reconstruction was used to reimplant the buttons. The patient was extubated on day 1, required a pacemaker on day 5, and went home on day 7. He is alive and well, 2 years postoperatively. Institutional review board approval was not required; written informed consent for publication of the study data was obtained from the patient.

**Figure 1:**
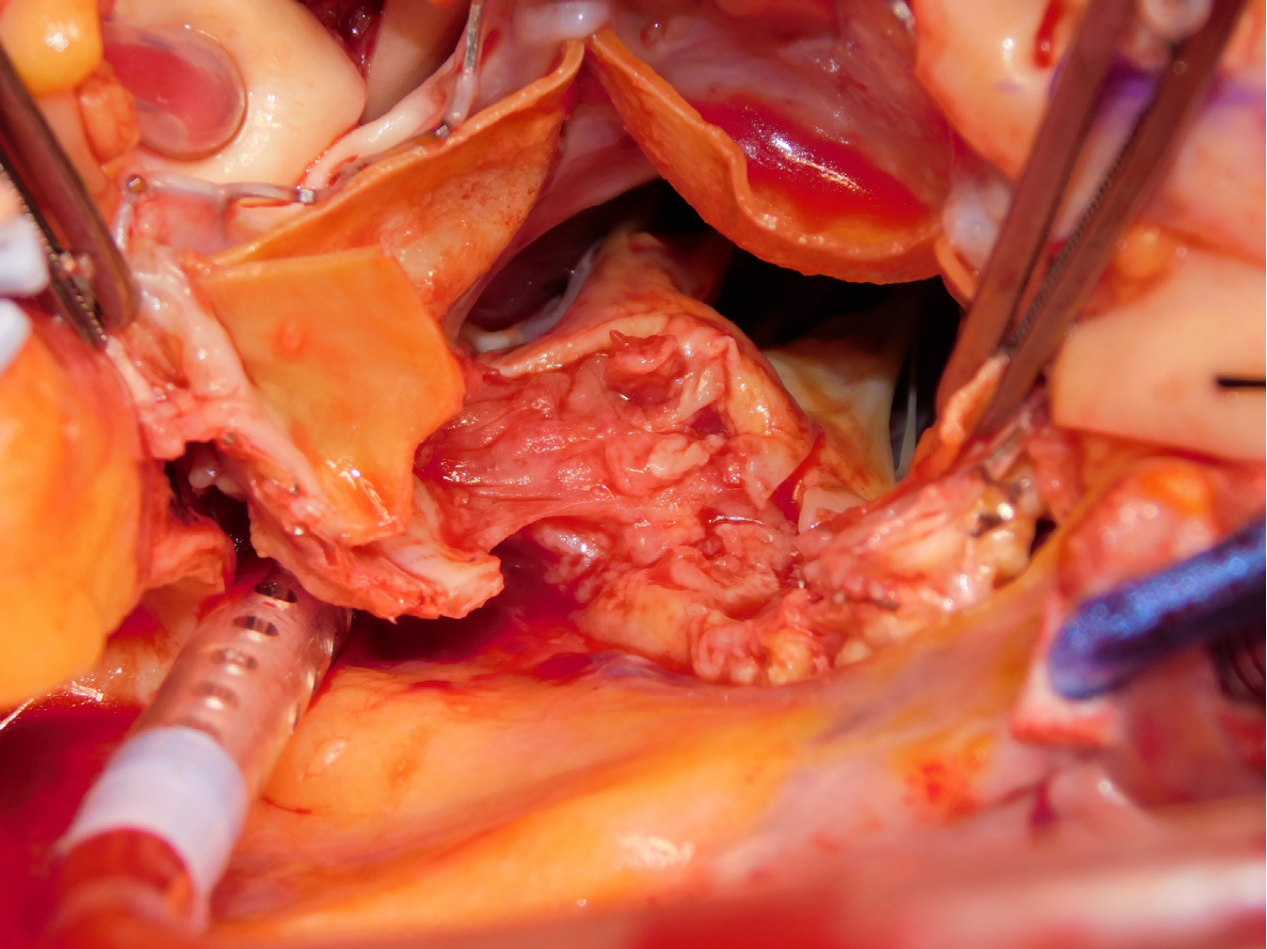
Extensive destruction of the aortomitral curtain.

**Figure 2:**
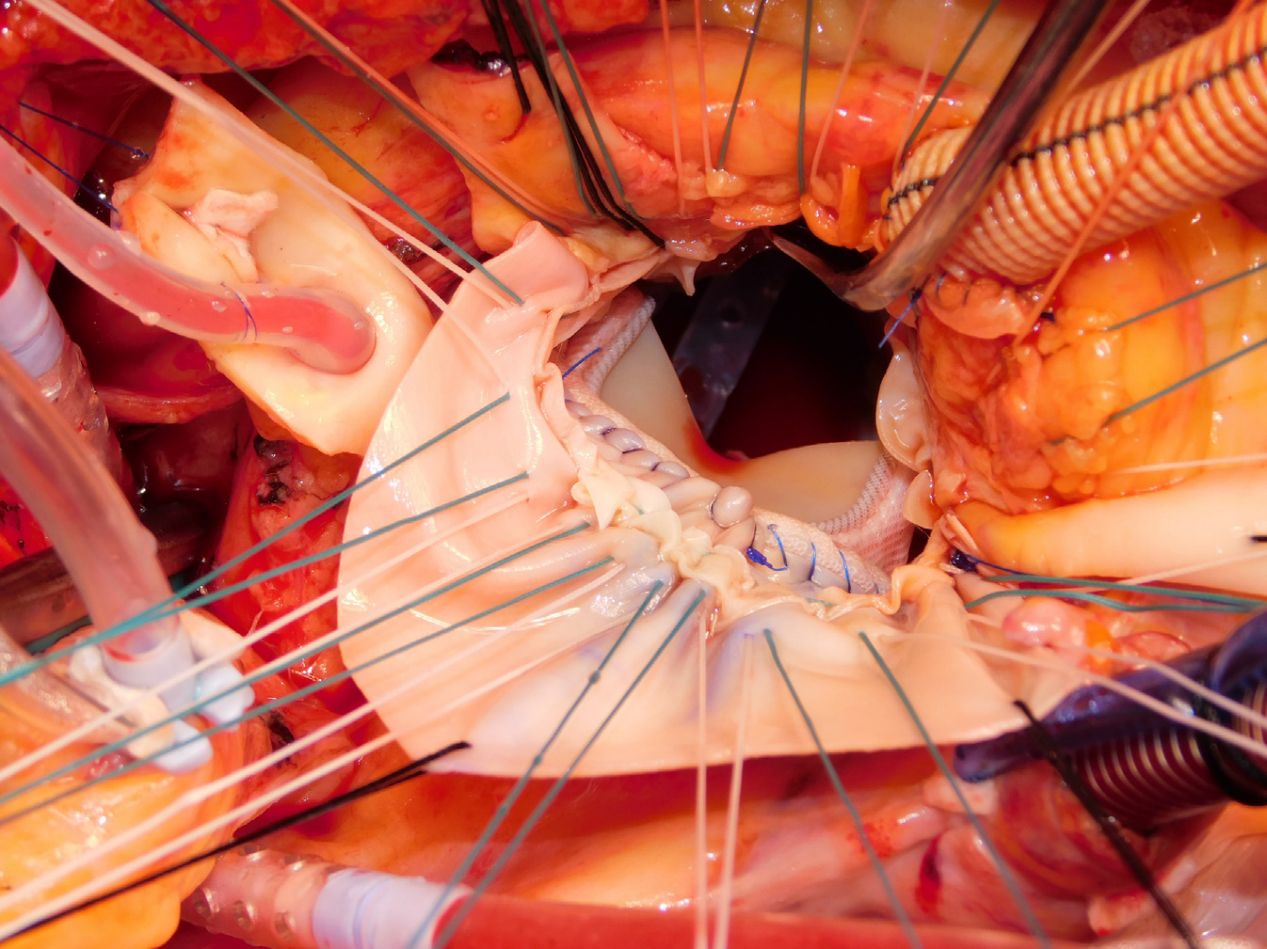
Prosthetic mitral valve in place with patch reconstruction of the aortomitral curtain, with adequate space for new neo-left ventricular outflow tract.

Below are 10 principles to achieve good outcome in the Commando procedure:

1. Radial debridement of the aortomitral curtain should be performed to reduce recurrence.
2. The sutures for the MV cover two-thirds of the annulus (trigone to trigone).
3. Do not oversize the MV, to allow space for the neo LVOT.
4. The anterior struts of the MV must align with the neo-LVOT.
5. The width of the patch corresponds to the distance between the trigones, with no tension.
6. Pay attention to the suture line of the patch (especially at trigones) because this is where it will bleed.
7. The gap between the aortic/mitral prosthesis should not be excessive, to avoid “rocking.”
8. Bathe graft material in rifampicin to reduce reinfection.^2^
9. Run an additional suture around the base of the root prosthesis to reduce bleeding.
10. Have a low threshold to perform modified Cabrol procedure to the buttons to reduce risk of kinking. Ringed expanded polytetrafluoroethylene grafts are less prone to compression.

### Webcast

You can watch a Webcast of this AATS meeting presentation by going to: https://www.aats.org/resources/tavr-valve-endocarditis-requir-7197.

**Figure:**
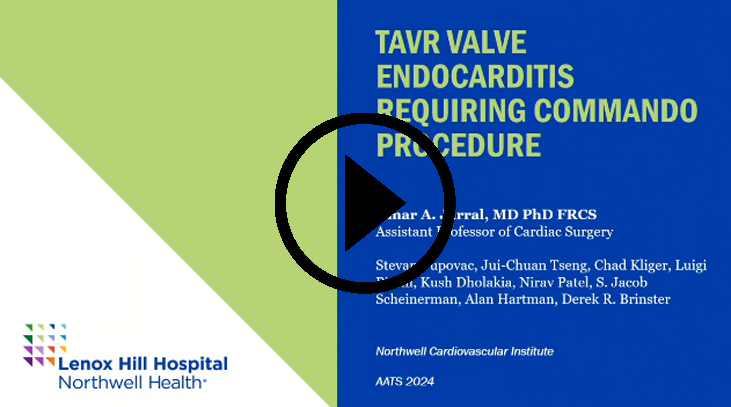

## Conflict of Interest Statement

The authors reported no conflicts of interest.

The *Journal* policy requires editors and reviewers to disclose conflicts of interest and to decline handling or reviewing manuscripts for which they may have a conflict of interest. The editors and reviewers of this article have no conflicts of interest.

## Supplementary Data

Video 1 Commando procedure for transcatheter aortic valve replacement valve endocarditis. Video available at: https://www.jtcvs.org/article/S2666-2507(24)00260-8/fulltext.
