# Supplementary figures and images for: Transcatheter aortic valve replacement valve endocarditis requiring Commando procedure
Source: JTCVS Tech. 2024 Jun 28;27:73–5. doi: 10.1016/j.xjtc.2024.06.011 (PMC11518893; doi:10.1016/j.xjtc.2024.06.011)

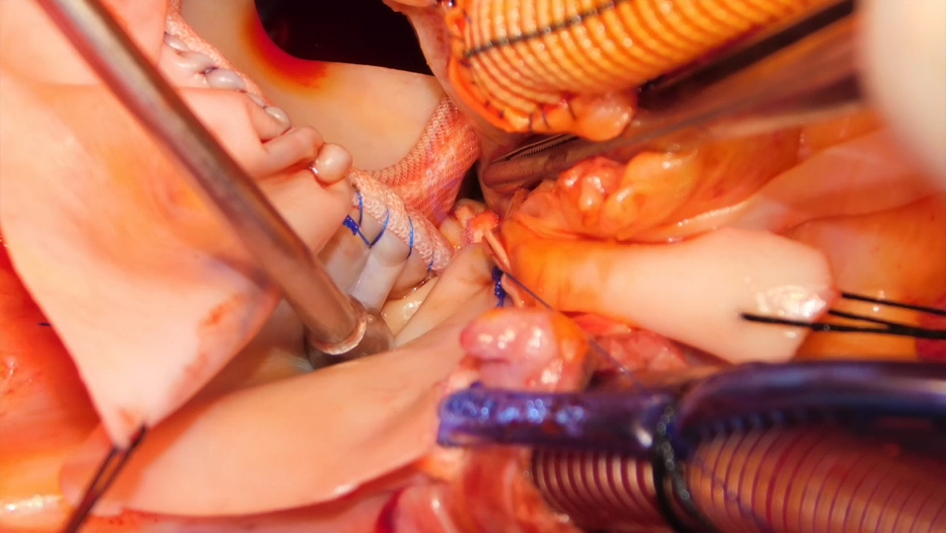

Supplement: Video 1 — Commando procedure for transcatheter aortic valve replacement valve endocarditis. Video available at: https://www.jtcvs.org/article/S2666-2507(24)00260-8/fulltext. [file fx3.jpg]
